# The Mediating Role of Internalized Stigma and Illness Knowledge in the Relationship Between Psychological Flexibility and Symptom Severity in Schizophrenia

**DOI:** 10.1007/s11126-025-10140-y

**Published:** 2025-04-04

**Authors:** Buket KOPARAL, İlknur KİRAZ AVCI

**Affiliations:** 1 Department of Psychiatry, Faculty of Medicine, Gazi University, Ankara, Turkey; 2 Rize State Hospital, Rize, Turkey

**Keywords:** Schizophrenia, Stigmatization, Self-esteem, Community mental health centers

## Abstract

Schizophrenia is a chronic mental illness that affects daily functioning and quality of life. Many patients experience internalized stigma, which worsens symptoms and quality of life. Psychological flexibility may help reduce stigma’s negative effects. This study explores how psychological flexibility, internalized stigma, and illness knowledge relate to symptom severity in schizophrenia. We hypothesized that internalized stigma mediates the link between psychological flexibility and symptoms, and that greater illness knowledge leads to lower stigma and better outcomes. This cross-sectional study included patients diagnosed with schizophrenia at a Community Mental Health Center (CMHC) in Turkey. 253 participants completed standardized scales of psychological flexibility(AAQ-II), internalized stigma(ISMI), knowledge about schizophrenia(KASQ), and symptom severity(PANSS). Mediation analysis was performed using the PROCESS macro for SPSS to assess the indirect effects of stigma and illness knowledge on symptom severity. Psychological flexibility was significantly associated with lower levels of internalized stigma (β = -1.046, *p* < 0.001). Internalized stigma mediated the relationship between psychological flexibility and symptom severity(β = 0.506, *p* < 0.001), whereas illness knowledge had a protective effect on symptom severity(β = -1.582, *p* < 0.001). However, illness knowledge did not significantly mediate the relationship between psychological flexibility and stigma. The findings highlight the critical role of psychological flexibility in mitigating the negative impact of internalized stigma, suggesting that interventions aimed at enhancing flexibility could improve clinical outcomes. Psychoeducation programs may further reduce symtom severity by increasing ilness knowledge. Future research should explore longitudinal interventions targeting stigma reduction and psychological flexibility to enhance functional recovery in schizophrenia.

## Introduction

Schizophrenia is a psychiatric disorder characterized by chronic or recurrent psychosis, accompanied by impairments in social and occupational functioning [1]. According to The Global Burden of Diseases, Injuries, and Risk Factors Study, schizophrenia is among the 15 most common causes of disability worldwide [2]. Despite advancements in pharmacological and psychosocial interventions, less than 15% of patients meet the criteria for clinical recovery [3]. The majority of individuals with schizophrenia experience significant impairments in quality of life and functional outcomes [1, 4].

Patients with schizophrenia exhibit impairments in self-care, interpersonal relationships, occupational functioning, and daily life activities not only during active psychotic episodes but also in periods of remission. This condition, defined as functional impairment, is influenced not only by illness-related factors but also by individual characteristics and environmental conditions [5]. Among the illness-related factors, negative symptoms and executive dysfunctions play a significant role, as they often emerge before the onset of the disorder and persist throughout its course [4, 6, 7]. Environmental factors primarily encompass psychosocial support, employment status, and socio-economic conditions [1, 8]**.** Additionally, individual characteristics such as personality traits, psychological flexibility, self-esteem, and self-stigmatization are considered to be associated with functional outcomes in individuals with schizophrenia [1, 9, 10].

Internalized stigma, characterized by the internalization of society's negative perceptions and prejudices by individuals, is particularly prevalent among those with mental disorders [11]. This phenomenon contributes to the development of maladaptive self-perceptions, diminished self-esteem, and impaired social functioning [12–14]. Empirical evidence from studies on individuals with schizophrenia has established a positive correlation between internalized stigma and clinical variables such as illness severity, duration of illness, and frequency of hospitalizations [15, 16]. Furthermore, internalized stigma has been associated with reduced adherence to treatment, an increased prevalence of comorbid psychiatric disorders, reduced qualiity of life and a heightened risk of suicidal behavior [17–19]. Studies on individuals with schizophrenia has also documented moderate levels of internalized stigma, with higher stigma scores being significantly associated with impaired social functioning and low functional recovery. [20, 21]. Given that internalized stigma adversely affects not only the individual but also their family, and considering its far-reaching detrimental consequences, addressing and mitigating stigma remains a critical priority in clinical and public health interventions.

Psychological flexibility is defined as an individual's capacity to adapt to changing circumstances, accept distressing thoughts and emotions without avoidance, and act in accordance with personal values [22]. A primary goal Acceptance and Commitment Therapy (ACT) is to enhance psychological flexibility, thereby enabling individuals to accept rather than struggle with challenging thoughts and emotions, ultimately facilitating a meaningful life. Research findings indicate that individuals with schizophrenia exhibit lower levels of psychological flexibility, which has been associated with greater severity of depressive symptoms, negative symptomatology [23, 24]. Patients who received ACT as an adjunct to standard treatments exhibited lower levels of functional impairment and a reduced frequency of hospitalizations [25, 26]. A study reported that no direct relationship was found between low psychological flexibility and the severity of psychosis; however, this association was better explained through the mediating effects of internalized stigma and social functioning [27]. A more detailed examination of the factors influencing psychological flexibility, along with targeted interventions in this domain, may contribute to reducing disease severity and improving patients' ability to cope more effectively with the impact of schizophrenia.

Based on existing literature, we hypothesize that higher levels of internalized stigma will be positively associated with greater psychotic symptom severity, whereas greater illness knowledge will be linked to lower internalized stigma and reduced symptom severity. Furthermore, we expect psychological flexibility to be inversely related to internalized stigma, as individuals with higher psychological flexibility may demonstrate greater resilience against self-stigmatization. Additionally, we propose that the relationship between psychological flexibility and symptom severity will be mediated by internalized stigma and illness knowledge. This study advances the field by offering a more comprehensive understanding of the complex interplay between psychological flexibility, internalized stigma, illness knowledge, and symptom severity. The findings have both theoretical and practical implications, suggesting that ACT-based interventions and psychoeducation tailored to reduce stigma could play a crucial role in schizophrenia treatment.

## Materials and Methods

### Participants and Procedure

A priori power analysis was conducted using G*Power 3.1.9.7 to determine the minimum sample size necessary for testing the study hypotheses. The analysis indicated that a sample size of N = 98 would be required to achieve 80% power for detecting a medium effect size at a significance level of α = 0.05 for F-tests. Given that the obtained sample size was N = 253, it is deemed sufficiently large to test the study hypotheses robustly.

This study is designed cross-sectionally. The study sample consisted of patients followed up at Rize Community Mental Health Center (RCMCH) in Turkey between June 2024 and November 2024, with diagnoses of schizophrenia according to ICD-10 criteria [28]. During the participant recruitment process, patients who attended the RCMCH for routine follow-up appointments were screened, and those meeting the inclusion criteria were invited to participate in the study after providing informed consent. While the participants were at different stages of their illness, all were in remission at the time of data collection. The inclusion criteria were as follows: (1) being between 18 and 65 years of age, (2) meeting the diagnostic criteria for schizophrenia according to ICD-10 and (3) providing consent to participate in the study. The exclusion criteria were: (1) having additional psychiatric diagnosis of mental retardation, substance/alcohol use disorders (2) having neurological diagnoses such as dementia or organic mental disorders, (3) being in a state of psychotic exacerbation, and (3) being illiterate or unable to speak Turkish. Additional psychiatric diagnoses—such as depression, anxiety disorders, or obsessive–compulsive disorder—were not excluded from the study.

All patients were informed about the study, and written informed consent was obtained from those who agreed to participate. Patients were assessed using the PANSS by the researcher İ.K.A. Subsequently, under the supervision of the same researcher, the patients completed the socio-demographic data form and other scales.

The necessary permissions for the study were obtained, and the study was approved by the Ethics Committee (Date: 11.06.2024 Approve number: 11) and was performed in accordance with the ethical standards of Declaration of Helsinki.

## Clinical Assesments

Socio-demographic form was created by researchers in order to assess patients’ age,education, marital and employement status, duration of illness, hospitalization history and current medications.

The Acceptance and Action Questionnaire-II (AAQ-II) was developed by Bond et al. in 2011. [29]. The Turkish adaptation of the scale was conducted in 2016 [30]. The scale demonstrated good internal consistency, with a mean Cronbach's alpha coefficient of 0.84 and a correlation coefficient of 0.85. The AAQ-II consists of 7 items, all of which are reverse-scored. The total scores range from 7 to 49, with higher scores indicating greater experiential avoidance and lower psychological flexibility.

The Knowledge About Schizophrenia Questionnaire (KASQ) is a 25-item self-report scale developed by Ascher-Svanum [31]. It includes questions related to the prevalence, etiology, course, and prognosis of schizophrenia, as well as pharmacological treatments and their side effects, non-pharmacological interventions, stress factors, and legal processes. The Turkish validity and reliability study was conducted by Atalan et al. [32]. Scores on the questionnaire range from 0 to 25, with higher scores indicating greater knowledge about schizophrenia. The scale demonstrated good reliability, with a Cronbach's alpha coefficient of 0.80 and a correlation coefficient of 0.775.

The Internalized Stigma of Mental Illness Scale (ISMI) was developed by Ritsher et al. in 2003 [33], Turkish validity and reliability study was conducted in 2007 [34]. ISMI is a 29-item self-report scale designed to assess internalized stigma. The total ISMI score ranges from 4 to 91, with higher scores indicating more severe levels of internalized stigma experienced by the individual.

The Positive and Negative Syndrome Scale (PANSS) is a clinician-administered, 30-item rating scale. It was developed by Kay, evaluates the severity of positive symptoms (PANSS-P), negative symptoms (PANSS-N), and general psychopathological symptoms [35]. Total scores on the PANSS range from 30 to 210, with higher scores reflecting greater severity of psychopathology. Turkish validity and reliability study was conducted [36].

## Statistics

All basic statistical analyses, including frequency analysis, mean, standard deviation, correlation and reliability, were performed using SPSS for Windows version 25.0 (SPSS Inc., Chicago, IL). To assess the distribution of the data, skewness and kurtosis values were examined. Values within the range of −2 to + 2 were considered indicative of a normal distribution. Additionally, visual inspection using histograms and Q-Q plots confirmed the normality of the data. Based on these evaluations, the distribution of the data was deemed appropriate for further analysis.

IBM SPSS Amos Graphics was utilized to assess the model fit for all scales. To investigate differences in UBQ scores across gender, social status, working status, and school year, independent-sample t-tests and one-way ANOVA were conducted.

Mediation analyses were performed using the PROCESS macro for SPSS (Model 6; Hayes, 2018) to evaluate the mediating effects of ISMI and KASQ on the relationship between AAQ and PANNS Additionally, the interaction effect of ISMI and KASQ on this relationship was analyzed. The statistical significance of the mediating variables was assessed using a bootstrapping method with 5,000 resamples. A 95% confidence interval that did not include zero was considered evidence of a significant indirect effect [37].

## Results

### Preliminary Analyses

The socio-demographic data of the participants are presented in Table 1. The study sample consisted of 253 participants with a median age of 46 years (range: 22–73 years) and a mean age of 46.98 ± 11.03 years. The majority of the participants were male (72.3%), while female participants accounted for 27.7% of the sample. In terms of educational status, 50.2% of the participants were literate without formal education, 22.9% had completed primary or secondary education, and 26.9% had attained a high school diploma or higher education.

**Table 1:** Demographic and characteristics of the sample

	*Md (Min–Max)*	*M * ± * SD*
***Age***	46 (22–73)	46.98 ± 11.03
	**n**	**%**
**Gender**		
Female	70	27.7
Male	183	72.3
***Education***		
Literate	127	50.2
Primary and Secondary school	58	22.9
High School and above	68	26.9
***Working Status***		
Unemployed	135	53.4
Employed	36	14.2
Retired	82	32.4
***Marital status***		
Married	78	30.8
Single	150	59.3
Divorced/widowed	25	9.9
***Living Arrangement***		
Single	14	5.5
With family	239	94.5
***Rehospitalization***		
Present	40	15.8
Absent	213	84.2
***Disease Type***		
Paranoid	148	58.5
Nonparanoid	105	41.5
***Pharmacological treatments*** ***Depot Antipsychotics***		
Yes	133	52.6
No	120	47.4
***Oral Antipsychotics***		
Yes	211	83.4
No	42	16.6
***Mood Stabilizers***		
Yes	69	27.3
No	184	72.7
***Antidepressants***		
Yes	59	23.3
No	194	76.7
***Clozapine***		
Yes	45	17.8
No	208	82.2

*Md* = Median*, Min* = Minimum*, Max* = Maximum*, M* = Mean, *SD* = Standard deviation

Regarding employment status, more than half of the participants (53.4%) were unemployed, while 14.2% were engaged in active employment, and 32.4% were retired due to disability. Marital status data indicated that 59.3% of the participants were single, 30.8% were married, 9.9% were divorced or widowed. The majority of the participants (94.5%) had lived with their families, whereas 5.5% lived alone. A family history of psychiatric illness was reported by 36.8% of the participants, whereas 63.2% had no such history. Hospitalization history revealed that 77.9% of the participants had been hospitalized at least once for psychiatric treatment, with 15.8% experiencing rehospitalization.

Clinically, 58.5% of participants were diagnosed with the paranoid subtype of schizophrenia, whereas 41.5% had a non-paranoid subtype. In terms of pharmacological treatment, 52.6% of the participants were receiving depot antipsychotic medication, while 47.4% were not. Oral antipsychotics were more commonly prescribed, with 83.4% of the sample using them. Additionally, 27.3% of participants were receiving mood stabilizers, 23.3% were prescribed antidepressants, and 17.8% were treated with clozapine.

The Table 2 presents descriptive statistics for the scales and correlations of variables.The PANSS has a median score of 57(range: 34–107) and a mean of 59.47 ± 15.64 Among its subscales, the PANNSS-P shows a median of 63 (range: 36–106) and a mean of 64.52 ± 11.94, the PANNS-N has a median of 11 (range: 8–24) and a mean of 12.74 ± 4.01, and the General Psychopathology subscale displays a median of 16 (range: 8–28) with a mean of 16.14 ± 5.13. For the ISMI, the median score is 29 (range: 18–58), with a mean of 30.58 ± 8.13. The AAQ has a median of 21 (range: 7–41) and a mean of 21.17 ± 8.67. Lastly, the KASQ scale shows a median score of 12 (range: 3–21) with a mean of 11.6 ± 4.13.

**Table 2:** Descriptive Statistics and correlation matrix of study variables

	*Md (Min–Max)*	*M * ± * SD*	1	2	3	4	5	6	7
1. PANNS	57 (34–107)	59.47 ± 15.64	1	0.823^**^	0.870^**^	0.970^**^	0.496^**^	0.395^**^	–0.428^**^
2. PANNS-P	63 (36–106)	64.52 ± 11.94	0.823	1	0.53	0.756	0.351	0.326	–0.231
3. PANNS-N	11 (8–24)	12.74 ± 4.01	0.870^**^	0.530^**^	1	0.782^**^	0.458^**^	0.319^**^	–0.424^**^
4. General psychopathology	16 (8–28)	16.14 ± 5.13	0.970^**^	0.756^**^	0.782^**^	1	0.492^**^	0.397^**^	–0.442^**^
5. ISMI	29 (18–58)	30.58 ± 8.13	0.496^**^	0.351^**^	0.458^**^	0.492^**^	1	0.760^**^	–0.041
6. AAQ	21 (7–41)	21.17 ± 8.67	0.395	0.326	0.319	0.397	0.76	1	0.048
7. KASQ	12 (3–21)	11.6 ± 4.13	–0.428	–0.231	–0.424	–0.442	–0.041	0.048	1

Note. *p < 0.05, ** p < 0.01, Md: Median, Min:Minimum, Max:Maximum, M: Mean, SD: Standard deviation PANNS: Positive and Negative Syndrome Scale, ISMI: Internalized Stigma of Mental Illness Scale, AAQ: Acceptance and Action Questionnaire, KASQ: Knowledge About Schizophrenia Scale

## Primary Analyses

**Fig. 1:**
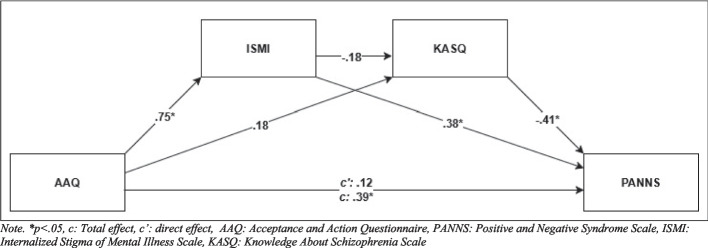
The estimated mediators model. Note. *p < *0.05, c: Total effect, c’: direct effect, AAQ: Acceptance and Action Questionnaire, PANNS: Positive and Negative Syndrome Scale, ISMI: Internalized Stigma of Mental Illness Scale, KASQ: Knowledge About Schizophrenia Scale*

The analysis was conducted to examine whether ISMI and KASQ mediate the relationship between the AAQ and the dependent variable PANNS (Table 3, Fig. 1). The constant represents the baseline value of the dependent variable when all predictors (AAQ and ISMI) are zero (b = 42.378, p < 0.001). The relationship between the independent variable (AAQ) and the mediator (ISMI) is statistically significant (b = 1.046, p < 0.001). The relationship between AAQ and the second mediator (KASQ) is non-significant (p > 0.05). The path from ISMI to PANNS is significant (b = 0.506, p < 0.001), indicating that higher ISMI scores are associated with higher PANNS scores. Conversely, KASQ has a significant negative effect on PANNS (b = −1.582, p < 0.001), showing that higher KASQ scores are linked to lower PANNS scores. The total effect represents the direct relationship between AAQ and the dependent variable without accounting for the mediators (ISMI and KASQ) (b = 0.712, p < 0.001). The coefficient has a significant positive effect on PANNS. The direct effect represents the relationship between AAQ and the dependent variable (PANNS) after accounting for the mediators (ISMI and KASQ). The non-significant p-value (p = 0.106) indicates that AAQ does not have a direct effect on the dependent variable when ISMI and KASQ are included in the model. The influence of AAQ on the dependent variable operates through ISMI and KASQ, pointing to full mediation. The path from ISMI to KASQ is also non-significant (b = −0.064, p = 0.057). The indirect effect of AAQ on PANNS through ISMI is significant (b = 0.293, CI [0.135, 0.418]), while the indirect effect through KASQ is non-significant (b = −0.142, CI [−0.307, 0.002]). The indirect effect involving both mediators (AAQ → ISMI → KASQ → PANNS) is non-significant (b = 0.106, SE = 0.058, CI [−0.003, 0.124]). Overall, the results highlight that ISMI significantly mediates the relationship between AAQ and PANNS, with KASQ showing a protective effect on PANNS but playing a less prominent mediating role.

**Table 3:** Results of Mediators Analysis

Effects	Coefficient	*SE*	*t*	*p*	95% CI (LL-UL)
Constant	13.822	1.5719	8.793	0.000*	(10.726, 16.918)
AAQ → ISMI (a_1_)	1.046	0.057	18.513	0.000*	(0.934, 1.157)
AAQ → KASQ (a_2_)	0.089	0.046	1.955	0.052	(–0.001, 0.180)
ISMI → PANNS (b_1_)	0.506	0.098	5.158	0.000*	(0.313, 0.699)
KASQ → PANNS (b_2_)	–1.582	0.185	−8.575	0.000*	(–1.945, −1.218)
AAQ → PANNS (total effect) (c)	0.712	0.105	6.805	0.000*	(0.506, 0.918)
AAQ → PANNS (c') (direct effect)	0.219	0.135	1.621	0.106	(–0.047, 0.485)
ISMI → KASQ (d_21_)	–0.064	0.033	–1.914	0.057	(–0.129, 0.002)
Indirect effect (a_1_*b_1_)	0.293	0.073	–	*	(0.135, 0.418)
Indirect effect (a_1_*b_2_)	–	0.077	–	–	(–0.307, 0.002)
Indirect effect (a_1_*d_21_*b_2_)	–	0.058	–	–	(–0.003, 0.124)
*R: 0.646, R*^*2*^*:0.4128, F* = *59.688, p* = < *0.001*					

*p < 0.05, SE: Standard Error, LL: Lower Level, UP: Upper Level, CI: Confidence Interval, PANNS: Positive and Negative Syndrome Scale, ISMI: Internalized Stigma of Mental Illness Scale, AAQ: Acceptance and Action Questionnaire, KASQ: Knowledge About Schizophrenia Scale

## Network Analysis

**Fig. 2:**
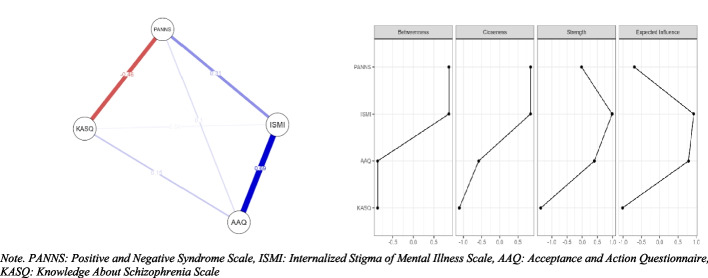
Network structure (left) and centrality indices (right). *Note. PANNS: Positive and Negative Syndrome Scale, ISMI: Internalized Stigma of Mental Illness Scale, AAQ: Acceptance and Action Questionnaire, KASQ: Knowledge About Schizophrenia Scale*

With the network analysis, we aimed to identify the data better, make predictions about the direction and strength of the relationships between the items. JASP 0.14.1 software was used for network analysis (see Fig. 2).

Table 4 presents the key characteristics of the network analysis. The constructed network comprises four nodes and six non-zero edges, indicating that all possible connections between the nodes are present. The calculated sparsity value of 0.00 confirms that the network is fully connected.

**Table 4:** Centrality measures per variable

		Network							
Variable		Betweenness		Closeness		Strength		Expected influence	
AAQ		–0.866		–0.579		0.399		0.786	
ISMI		0.866		0.845		0.989		0.928	
KASQ		–0.866		–1.111		–1.365		–1.018	
PANNS		0.866		0.845		–0.022		–0.696	

An evaluation of centrality measures highlights the distinct roles of the variables within the network. ISMI emerges as the most central variable, exhibiting high betweenness (0.866), closeness (0.845), strength (0.989), and expected influence (0.928), suggesting a strong influence and high connectivity within the network. In contrast, KASQ demonstrates consistently negative centrality values across all measures, including betweenness (−0.866), closeness (−1.111), strength (−1.365), and expected influence (−1.018), indicating a peripheral role with limited influence on other variables.

AAQ-II displays moderate positive strength (0.399) and expected influence (0.786); however, it has lower betweenness (−0.866) and closeness (−0.579), suggesting a relatively focused but less central role in the network. Meanwhile, PANSS exhibits high betweenness (0.866) and closeness (0.845) but lower strength (−0.022) and expected influence (−0.696), indicating that while it functions as a bridge connecting variables, it does not exert substantial direct influence within the network.

## Discussion

In this study, which investigates the associations between psychological flexibility, internalized stigma, illness knowledge, and symptom severity in individuals with schizophrenia, our findings indicate that while no direct relationship exists between psychological flexibility and symptom severity, internalized stigma and illness knowledge function as mediating variables in this association. These results contribute to the growing body of literature elucidating the psychosocial mechanisms underlying symptom severity in schizophrenia and underscore the critical role of psychological flexibility in shaping individuals’ cognitive and emotional responses to their illness. Notably, internalized stigma exhibited a positive correlation with symptom severity, whereas greater illness knowledge was associated with a protective effect, mitigating symptom severity.

An analysis of the clinical scale results revealed that the mean level of internalized stigma among the participants was 30.58 ± 8.13, indicating a moderate level of self-stigmatization and the mean PANSS score was 59.47 ± 15.64, indicating that while the majority of patients were clinically stable, they exhibited mild psychopathology (Table 2). A positive association was identified between illness severity and self-stigma levels.  In a study conducted by Vrbova et al. [15], individuals with schizophrenia exhibited significantly higher levels of self-stigmatization, with self-stigma positively correlating not only with illness severity but also with hospitalization frequency and treatment duration [15]**.** In another study conducted in 2018, patients were found to have a high level of internalized stigma, and the duration of illness was correlated with the degree of internalized stigma [38]. The relatively lower levels of self-stigma observed in our study may be attributable to sample characteristics. Patients who attend regular follow-ups may have lower disease severity and, consequently, lower levels of internalized stigma. Moreover, regular follow-up may contribute to a more stable course of illness, while the psychoeducational programs and informational sessions provided at the Community Mental Health Center (CMHC) may have further contributed to the reduction in stigma levels.

In our study, no direct significant association was found between illness-related knowledge and internalized stigma (Table 3). Previous interventions aimed at reducing internalized stigma have primarily focused on defining stigma, highlighting its negative consequences, and implementing strategies to mitigate its effects [39, 40]. However, the relationship between illness knowledge and internalized stigma remains underexplored in the literatüre, with conflicting findings. Some studies have suggested a potential link between these variables; for example, Singh et al. reported that greater illness knowledge was associated with lower internalized stigma [41]. Similarly, a 2012 study found that psychoeducation not only improved patients' understanding of their illness but also enhanced treatment adherence and reduced internalized stigma [42]. Conversely, Alhadidi et al. did not observe a significant relationship between schizophrenia knowledge and internalized stigma, which aligns with our findings [43]. These discrepancies may stem from variability in psychoeducational interventions, as some programs emphasize interactive engagement and stigma reduction, while others provide only general illness-related information, which may not be sufficient to change deeply ingrained beliefs. Despite the low levels of illness-related knowledge in our study sample, the relatively mild levels of internalized stigma and illness severity may be attributed to the stability of the patients and their access to social support mechanisms. Additionally, the lack of a significant association between illness knowledge and internalized stigma in our study suggests that merely increasing patients’ knowledge about their condition may not be sufficient to effectively reduce stigma. Internalized stigma is a multifaced issue shaped by multiple factors, including treatment adherence, coping mechanisms, insight, and self-efficacy, as well as broader social influences such as public awareness and psychosocial interventions [39, 44]. Additionally, cultural and environmental factors may influence the relationship between illness knowledge and stigma, as societies with stronger stigma surrounding mental illness may require broader social interventions rather than individual psychoeducation alone. Measurement differences across studies may also contribute to inconsistent findings, as different tools may capture varying dimensions of internalized stigma .Therefore, efforts to mitigate internalized stigma should not only focus on enhancing patients' knowledge about their illness but also include strategies aimed at improving self-efficacy and psychological flexibility, strengthening social support mechanisms, and implementing interventions to challenge and change stigmatizing societal attitudes.

Interestingly, our findings revealed a negative association between illness knowledge and symptom severity, suggesting that individuals with greater illness-related knowledge exhibit lower symptom severity (Table 3). While illness knowledge does not appear to have a direct effect on internalized stigma, its significant relationship with symptom severity underscores its potential role in facilitating better disease management through enhanced understanding and treatment adherence. This highlights the necessity of implementing targeted educational interventions, particularly for patients with poor adherence, limited insight, and more severe symptomatology. Notably, previous research has demonstrated that increased awareness of illness and treatment processes contributes to improved adherence and insight, thereby corroborating our findings [45, 46].

Our findings indicate that individuals with schizophrenia exhibit low levels of psychological flexibility, which is significantly associated with internalized stigma. Psychological flexibility refers to an individual’s capacity to adapt to changing environmental conditions and effectively cope with distressing emotions [22]. Previous studies have demonstrated that individuals with schizophrenia exhibit markedly lower psychological flexibility compared to the general population, and this has been linked to symptom severity, cognitive functions, and overall functional outcomes [23, 24].In our study, lower psychological flexibility was found to be significantly associated with higher levels of internalized stigma, whereas no direct relationship was observed between psychological flexibility and either symptom severity or illness knowledge. The strong association between psychological flexibility and internalized stigma suggests that individuals with schizophrenia may be more prone to adopting negative beliefs about their illness and internalizing societal prejudices. Internalized stigma leads individuals to perceive themselves as devalued and socially excluded due to their illness, thereby reducing treatment adherence, increasing social isolation, and negatively impacting overall functioning [11, 14, 15]. Individuals with lower psychological flexibility may struggle to cope with negative illness-related thoughts, potentially reinforcing their internalized stigma. Intervention strategies for addressing internalized stigma are primarily grounded in two theoretical approaches. The first approach seeks to modify self-stigmatizing beliefs and attitudes, whereas the second approach emphasizes the acceptance of stigmatizing stereotypes without directly challenging them, aiming to enhance stigma-coping mechanisms through improvements in self-esteem, empowerment, and help-seeking behaviors [47]. ACT predominantly aligns with the latter approach, demonstrating its efficacy by fostering psychological flexibility, emotional regulation and adaptive coping strategies in individuals experiencing internalized stigma. Facilitating individuals' acceptance of stigmatizing thoughts rather than engaging in cognitive struggle, while promoting engagement with present experiences independent of past negative encounters or societal labels, may contribute to the reduction of internalized stigma within the framework of ACT. Additionally, encouraging a transition from a disease-centered identity toward the pursuit of meaningful goals, alongside the integration of self-compassion-enhancing techniques, may further support efforts to mitigate internalized stigma.Literature suggests that ACT have been effective in reducing internalized stigma and improving psychosocial functioning in individuals with schizophrenia [25, 48–50].

Our mediation model further supports these findings, revealing that the effect of psychological flexibility on symptom severity is not direct but is instead mediated by internalized stigma. The absence of a direct relationship between psychological flexibility and symptom severity aligns with prior research. For instance, a study by González-Menéndez et al. (2021) found that psychological flexibility was not directly associated with the severity of psychotic symptoms, but rather, this association was better explained through internalized stigma and social functioning [27]. This suggests that psychological flexibility may not exert a direct effect on illness severity but may instead influence symptomatology through psychosocial factors such as stigma. Mechanisms through which internalized stigma exacerbates symptom severity may include nonadherence to treatment, reduced self-efficacy, and increased psychological distress [16–18]. Given these findings, interventions aimed at reducing symptom severity in schizophrenia should not solely focus on pharmacological treatments but also incorporate psychosocial strategies that enhance psychological flexibility and mitigate internalized stigma. Future research should further investigate the interplay between psychological flexibility and internalized stigma to develop targeted interventions that foster greater engagement in treatment and improve clinical outcomes for individuals with schizophrenia.

One of the key strengths of this study is its comprehensive examination of the relationships between psychological flexibility, internalized stigma, illness knowledge, and symptom severity, offering a multidimensional perspective on the impact of psychosocial variables on the course of schizophrenia, rather than analyzing these factors in isolation as seen in previous research. Notably, the finding that illness knowledge does not directly influence internalized stigma, while psychological flexibility exerts an indirect effect on symptom severity through internalized stigma, underscores the need for interventions that go beyond merely increasing illness-related knowledge through psychoeducation. Instead, it highlights the significance of intervention techniques aimed at enhancing psychological flexibility—such as ACT—making a valuable contribution to the existing literature. This research incorporates several methodological strengths that enhance the reliability and generalizability of the findings. A large, well-defined clinical sample (N = 253), providing greater statistical power compared to previous smaller-scale studies [21, 24, 27]. Using of validated psychometric tools (AAQ-II, ISMI, KASQ, PANSS), ensuring robust and reliable measurement of key variables.

However, certain limitations should also be acknowledged. The cross-sectional design restricts the ability to infer causal relationships between variables, highlighting the need for longitudinal studies to further validate the findings. Additionally, the sample consists exclusively of patients who regularly attend a CMHC, which may limit the generalizability of the results, as individuals with more severe illness and restricted access to healthcare services may not be adequately represented. Moreover, the non-significant relationship between illness knowledge and internalized stigma suggests the need for future research to explore additional factors (e.g., level of insight, treatment adherence) that may better explain this interaction. Despite these limitations, this study provides valuable insights into the role of psychosocial factors in symptom severity among individuals with schizophrenia.

## Conclusion

This study provides valuable insights into the complex interplay between psychological flexibility, internalized stigma, illness knowledge, and symptom severity in individuals with schizophrenia. Our findings suggest that while psychological flexibility does not exert a direct influence on symptom severity, its impact is mediated by internalized stigma, emphasizing the importance of addressing stigma in clinical interventions. Moreover, the negative association between illness knowledge and symptom severity highlights the potential role of psychoeducational interventions in improving patient outcomes. These results underscore the necessity of incorporating psychosocial strategies, alongside pharmacological treatments, to enhance treatment adherence, foster psychological flexibility, and mitigate internalized stigma in individuals with schizophrenia. Future research should explore tailored interventions that enhance psychological flexibility and address internalized stigma, particularly within early-stage and high-risk populations. Additionally, longitudinal studies examining the long-term effects of stigma reduction and psychoeducation on symptom severity and treatment adherence would further clarify the mechanisms underlying these associations.

## Data Availability

Data will be available on request.
